# Contemporary Management of Male Anterior Urethral Strictures by Reconstructive Urology Experts—Results from an International Survey among ESGURS Members

**DOI:** 10.3390/jcm11092353

**Published:** 2022-04-22

**Authors:** Felix Campos-Juanatey, Enrique Fes-Ascanio, Jan Adamowicz, Fabio Castiglione, Andrea Cocci, Guglielmo Mantica, Clemens Rosenbaum, Wesley Verla, Malte W. Vetterlein, Marjan Waterloos, Luis A. Kluth, on behalf of the Trauma and Reconstructive Urology Working Party of the European Association of Urology Young Academic Urologists (EAU YAU)

**Affiliations:** 1 Andrology and Reconstructive Urology Unit, Marques de Valdecilla University Hospital, Institute of Research Valdecilla (IDIVAL), 39011 Santander, Spain; 2 Urology Unit, Can Misses Hospital, 07800 Ibiza, Spain; enriquefes@gmail.com; 3 Department of Regenerative Medicine, Collegium Medicum, Nicolaus Copernicus University, 85-067 Bydgoszcz, Poland; adamowicz.jz@gmail.com; 4 Department of Urology, University College London Hospital, London W1G 8PH, UK; dr.castiglione.fabio@gmail.com; 5 Department of Urology and Andrology, University of Florence, 50100 Florence, Italy; cocci.andrea@gmail.com; 6 Department of Urology, Policlinico San Martino Hospital, University of Genova, 16132 Genova, Italy; guglielmo.mantica@gmail.com; 7 Department of Urology, Asklepios Klinik Barmbek, 22308 Hamburg, Germany; rosenbaumclemens@googlemail.com; 8 Department of Urology, Ghent University Hospital, 9000 Ghent, Belgium; wesley.verla@uzgent.be; 9 Department of Urology, University Medical Center Hamburg-Eppendorf, 20246 Hamburg, Germany; m.vetterlein@uke.de; 10 Department of Urology, AZ Maria Middelares Ghent, Division of Reconstructive Urology, Ghent University Hospital, 9000 Ghent, Belgium; marjan.waterloos@azmmsj.be; 11 Department of Urology, University Hospital Frankfurt, Goethe University Frankfurt am Main, 60590 Frankfurt am Main, Germany; luis.kluth@kgu.de

**Keywords:** anterior urethral strictures, endoscopic surgical procedures, health care surveys, reconstructive surgical procedures, surgical flaps, tissue grafts, urologic surgical procedures

## Abstract

Assessment of anterior urethral stricture (US) management of European urology experts is relevant to evaluate the quality of care given to the patients and plan future educational interventions. We assessed the practice patterns of the management of adult male anterior US among reconstructive urology experts from European countries. A 23-question online survey was conducted among European Association of Urology Section of Genito-Urinary Reconstructive Surgeons (ESGURS) members. A total of 88 invitations were sent by email at two different times (May and October 2019). Data were prospectively collected from May 2019 to December 2019. The response rate was 55.6%. Most of the responders were between 50 and 59 y.o. and mainly from University Public Teaching/Academic Hospitals. A total of 73.5% treated ≥20 patients/year with US. Retrograde urethrogram (RUG) was the commonest diagnostic tool, followed by uroflowmetry (UF) +/− post-void residual (PVR). Urethroplasty using grafts was the most frequent treatment (91.8%). Of responders, 55.3% performed >20 urethroplasties/year. Anastomotic urethroplasties were performed by 83.7%, skin flap repairs by 61.2%, perineal urethrostomy by 77.6% and non-transecting techniques by 63.3%. UF was the most common follow-up tool. Most of the responders considered urethroplasty as the primary option when indicated. Male anterior US among ESGURS members are treated mainly using urethroplasty graft procedures. RUG is preferred for diagnosis, and UF for follow-up.

## 1. Introduction

The management options for male urethral strictures (US) range from minimally invasive endoscopic interventions, successful in only carefully selected patients, to open urethroplasties, achieving excellent outcomes in most of the strictures [1,2]. Previously published evaluations evidence changes in trends on US treatment between different countries [3,4,5,6,7,8,9,10,11,12,13,14]. Investigating a group of subspecialized urologists could be relevant to evaluate if their practices are updated and evidence and guidelines based. Their responses could show differences with previous surveys: higher volume of surgeries, greater percentage of academic practices, tendency to open repairs and to use newer urethroplasty techniques. Furthermore, knowledge of current therapeutic choices could lead to design educational programs, if they seem required [13].

We aim to describe the practice patterns on diagnostic and therapeutic approaches for adult male anterior US among members of the European Association of Urology Section of Genito-Urinary Reconstructive Surgeons (ESGURS).

## 2. Materials and Methods

A non-validated questionnaire, based on previously published surveys conducted in individual countries was designed by the Trauma and Reconstructive Urology Working Party of the European Association of Urology (EAU) Young Academic Urologists (YAU). The final version included 23 items, evaluating demographic data and questions related to diagnosis, treatment, beliefs and opinions related to adult male anterior US management (Supplementary Material S1). The online version was hosted in the webpage of EAU and checked by the authors to ensure its accuracy and confirm the adequate functioning of the survey tool. A presentation of the project along with invitation letters were sent by email from EAU sections office to all members of ESGURS. Individual links to the online survey were included in each mail, directing to one single questionnaire per invitation, avoiding duplicated answers. The questionnaire was self-administered and anonymous. Initial mailing was in May 2019, with a second invitation letter (reminder) being sent in October 2019 to non-responders. Information was collected during an 8 month-period, between May and December 2019. The published Checklist for Reporting Results of Internet E-Surveys (CHERRIES) [15] was followed while conducting this study.

All answers were categorized and securely stored in an online database. For analysis, we considered all questionnaires, including those with incomplete information. Frequency tables for each question were extracted, summarizing the distribution of answers. Statistical analysis was performed using STATA 13.1 software for Mac (StataCorp, College Station, TX, USA).

## 3. Results

### 3.1. Descriptive Characteristics of Participating ESGURS Members

Of the 88 invitation letters sent to ESGURS members, all were received. In total, 49 of the approached members followed their individual link to the survey webpage, and all 49 completed the questionnaire. The response rate was 55.6%. Demographic data of the sample (age ranges, hospital type and level) is shown in Table 1.

Data on country of practice distribution is displayed in Table 2. In total, 87.8% of responders answered that there is a unit or person specially dedicated to urethral disease in their centres.

None of the urologists reported not treating any patients with US during the last year, while most of the urologists (73.5%) treated >20 patients per year; 26 urologists (55.3%) performed >20 urethroplasties yearly, while only 3 (6.4%) stated not performing them (see Table 3 for details).

### 3.2. Diagnostic and Follow-Up Strategies

Preferred diagnostic tools and follow-up methods are summarized in Table 4. While retrograde urethrography (RUG) is the commonest diagnostic technique, uroflowmetry (UF) is the most widespread follow-up tool. RUG is performed by a radiologist in 55.3% of answers, while a urologist is the one conducting the test in the remaining cases.

### 3.3. Management of Urethral Strictures

The full distribution of the different management options for anterior US is shown in Table 5.

#### 3.3.1. Endoscopic Techniques

Regarding endoscopic techniques, the most commonly performed was direct vision internal urethrotomy (DVIU) at 79.6%, followed by urethral dilation (UD) at 63.3%. The maximal length of stricture suitable for both endoscopic therapies was <1 cm for 24 urologists (49%), 8 (16.3%) answered <1.5 cm, and 12 (24.5%) < 2 cm. While performing these endoscopic procedures, 34 (69.4%) routinely used a guidewire or ureteric catheter to reference true urethral lumen, while 14 (28.6%) stated using them only in selected cases. These safety measures are never used by only 1 responder (2%). After both urethrotomies or dilatations, 19 responders (38.8) kept the urethral catheter 24 h, 21 (42.9%) < 3 days, 7 (14.3%) between 4–6 days, and 1 (2%) between 1–3 weeks: only 1 urologist (2%) did not routinely leave the urethral catheter, and no one maintained it for >3 weeks. The size of the urethral catheter to remain in place after DVIU or UD was 16F for 20 responders (42.6%), 14F for 11 (23.4%) and 18F for 9 (19.2%). Wider catheters were preferred by two urologists (4.3%), while narrow sizes were chosen by the same number of responders. Three colleagues (6.4%) claimed they did not have a preference for any catheter size.

#### 3.3.2. Urethroplasty Techniques

For bulbar strictures, dorsal graft urethroplasties and end-to-end anastomotic techniques are the most frequently described therapeutic approaches (Table 6). After urethroplasties, 30 responders (68.2%) routinely performed radiographic control images before or immediately after removing the urethral catheter, while 6 urologists (13.6%) did not at all; 8 responders (18.2%) claimed to do it not routinely but depending on each individual case.

### 3.4. Clinical Cases

The participants were asked about how they would manage two different anterior US patients. The first case presented was a 34 year-old uncircumcised male, with a 3.5 cm idiopathic bulbar US, complaining of poor stream and with maximum flow rate (Qmax) of 7 mL/s. Nearly half of responders 22 (46.8%) would offer him a dorsal augmentation urethroplasty using grafts, while 7 (15%) considered him a candidate for endoscopic management (see details in Table 7). The second scenario was a 24 year-old male, with 1 cm idiopathic proximal bulbar US, with two previous failed DVIUs (in the last two years), complaining of poor flow and with Qmax of 6 mL/s. In this case, end-to-end anastomotic urethroplasty would be the choice for 14 responders (30.4%), with only 3 urologists (6.6%) offering him endoscopic treatments (see details in Table 8). Participants were also asked about their beliefs in the management of US. The so-called “therapeutic ladder”, starting with minimally invasive procedures (UD, DVIU) and considering urethroplasty only after repeated failure of these procedures was supported by 12 urologists (25.5%). The remaining 35 responders (74.5%) considered urethroplasty as the primary option, when indicated.

### 3.5. Opinions

When asked about the need for referral centres for treatment of male anterior US disease, 45 of ESGURS member participants (95.7%) considered them as necessary, and 41 (87.2%) rated their specific training on management of US as adequate. Regarding training, 41 (87.2%) considered both theoretical and hands-on-courses as useful, while 4 (8.5%) preferred only hands-on ones. Only one considered the courses on this topic as useless, and one also preferred only theoretical lectures.

## 4. Discussion

Male US disease is a common and challenging health problem, increasing with the ageing of the population [11]. The estimated prevalence ranges between 229 and 627 cases per 100,000 adults [16]. Minimally invasive endoscopic treatments are the most common options for US, in both primary and recurrent setting [17]. They proved only to be curative for selected cases, with limited chances for definitive success when repeated more than two times [18,19,20]. These repetitions are not cost effective [20,21], but do not seem to affect the outcome of further urethral repairs [22,23]. Urethroplasty is the definitive treatment for most anterior US, with excellent outcomes in long-term follow-up [1,24], and should be offered as a first option when indicated [1,25]. A wide variety of techniques have been described for urethral repair, depending on strictures and patient characteristics [1,26].

Evaluation of practice patterns in US management started in 2005, with a mailed survey designed to assess pelvic fracture-related urethral injuries treatment among those practicing in the United Kingdom and Ireland [5]. Another mailed survey was conducted but focused on anterior US management among board certified urologists in the USA [6]. In both studies, an excess of endoscopic management was evidenced, proposing the non-familiarity with urethral surgery and limited knowledge of the literature on this topic as the reasons. Based on the original questionnaire used in the American study [6,7], national surveys on anterior US practices were conducted in the Netherlands [13], Italy [12] and Germany [27]. Recently, a most complete non-validated questionnaire was also used in Spain [14].

ESGURS group intends to bring together senior experts in the field of reconstructive urology and young urologists interested in reconstructive urological surgery. All members had their application form reviewed by ESGURS Board, requiring for acceptance a minimum of two peer-reviewed publications in the field of genitourinary reconstructive surgery, along with the written support of at least two ESGURS board members -certifying that ESGURS candidates are involved in clinical and academic activities within the area of genitourinary reconstructive surgery.

The response rate tends to be variable, depending on the targeted population. The original USA study, performed on a randomly selected sample of board-certified urologists, had a 34% response rate [6], in Spain was 21.7% with all members of the Spanish Urological Association being targeted [14], and in German response rate was 14.6% [27]. The highest response rate to date was achieved by Dutch and Italian studies (74% and 74.7%, respectively), but the first survey was conducted over all the urologists in the Netherlands—which has a small population—while the second was distributed between a randomly selected group of Italian urologists, with no information about how the authors selected them. We targeted all members of ESGURS group with an acceptable response rate (55.6%). Most of ESGURS responders work in academic/teaching hospitals (77.6%), similar to the Spanish survey (70%), and higher than in Italy (9.2%), the USA (10.7%), the Netherlands (18%) and Germany (20%).

The number of strictures managed by ESGURS urologists per year (73.5% > 20) is also higher when compared to other surveys: 38.7% in Spain, 30.1% in the Netherlands, 20% in Germany, 13.7% in the USA and 5.9% in Italy. Clearly this is because ESGURS members are all specialized in US. Likewise, the percentage of ESGURS urologists performing urethroplasties per year is higher (91.8%). Conversely, only 22.1% stated not performing urethroplasties in Spain, 77% in Netherland, 73.2% in Germany, 60.8% in Italy and 57.8% in the USA.

RUG and UF are the most common diagnostic tools, very similar to previous surveys, except in Italy where they use more frequently urethroscopy than RUG. For follow-up, the UF is the most routinely performed, as in all previous surveys, and according to recommended practices [2].

In previous surveys, the DVIU and UD were the most widespread treatment options, but among ESGURS members urethroplasties are the commonest techniques used. This is probably due to specialized characteristics of ESGURS members—working in academic high-volume centres where patients would be referred after endoscopic attempts—ESGURS members have preference for dorsal grafts (36.4%) versus ventral ones (29.6%) for bulbar strictures, which is similar to Spain. In other countries, the ventral location is the most widespread for graft augmentation. In line with previously published data, use of flaps is almost anecdotic for bulbar location.

The selection of patients suitable for endoscopic therapies seems adequate among ESGURS members, with 65.31% of responders using them on strictures <1.5 cm compared to Spain (84.4%), Italy (71.5%), the Netherlands (50.2%) or the USA (44.1%). ESGURS practitioners tend to use a guidewire during DVIU and leave a urethral catheter (14–18F) in place < 3 days (81.64%) as advocated by current evidence [28], which is opposite to Spain (13.8%). Conversely, 2% of ESGURS members keep urethral catheter after endoscopic procedures for 1–3 weeks, in line with 1.6% in Germany, but different from 8.2% in the USA and 38% in Spain.

As in previous surveys, two clinical cases were asked about the management of different situations. In a long (3.5 cm) bulbar stricture in a young patient, ESGURS responders would perform a graft augmentation, preferably dorsal, with only 14.9% offering endoscopic options. In other surveys, the minimally invasive treatment was chosen for a case like this by between 20.5 and 43.8% of responders. A second case asked about a young male with a short bulbar stricture, presented after two failed DVIUs. End-to-end anastomotic urethroplasty would be offered by most of responders (30.4%), as previously reported in another surveys, but the non-transecting technique was the first option for the 23.9%.

Most of ESGURS responders (74.5%) acknowledge that urethroplasty could be the first line therapy according to current evidence [28], instead of climbing a “therapeutic ladder” and performing open repairs only after endoscopic attempts. This is similar to the recent survey in Spain (70.9%), but in previous studies the commonest opinion was different, with only 21.3% supporting an urethroplasty as initial treatment in the USA (survey performed in 2002), 21% in the Netherlands, 26.2% in German and 33.8% in Italy. These data evidence that an appropriate management requires adequate education of the urological community, exposing the poor outcome of repeated endoscopic manoeuvres and current non-justification of “therapeutic ladder” theory, as a primary urethroplasty would lead to the best prognosis in certain patients and strictures [6,7,8,9,10,11,12,13,14,15,16,17,18,19,20,21,22,23,24,25,26,27,28].

Depending on the number of urethroplasties performed annually, the answers showed significant differences. Urologists performing a higher number of procedures are more prone to choosing urethroplasty as the first option in selected patients. This conclusion was also achieved in the American survey [6,7], and supports the need for high volume specialized centres. Teaching hospitals have urethral disease units more frequently than non-teaching, and in the German survey they are more likely to select open reconstructive treatments, instead of endoscopic therapies. Likewise, high volume surgeons use better diagnostic and follow-up tools, such as PROM questionnaires, and more accurate imaging studies in previously published studies. According to their specialization, ESGURS surgeons are also using new urethroplasty techniques (as non-transecting ones) in a significantly higher percentage than in other surveys. Most of ESGURS responders (95.7%) agreed with the need for referral centres for treatment of male anterior US disease. This was also suggested for both anterior [12,13] and posterior urethral injuries [5], but not asked directly in previous surveys, except in Spain (88.4%).

Many ESGURS urologists performed control images before or immediately after removing the urethral catheter in urethroplasties, some routinely (68.2%), others depending on the specific case (18.2%). It seems to be important to assess for urinary extravasation to avoid ensuing complications including peri-urethral inflammation, abscess formation and fistulation [2,3,4,5,6,7,8,9,10,11,12,13,14,15,16,17,18,19,20,21,22,23,24,25,26,27,28].

This survey could present some possible limitations that should be discussed. One could be related to different access to email and to a computer between members, leading to age bias. Another source of bias could be related with differences in time between the responders’ training and our study period, as certainly these years of practice may be important to correlate with diagnostic and therapeutic choices. Such information—years of practice since completing training—is not available, but as the distribution of ages of responders is uniform, it is unlikely that more young urologists were selectively targeted and therefore biased the obtained results. Another limitation could be the response rate (55.6%), but this is among the higher range for internet-based surveys. As we have mentioned before, the reply rate to surveys tends to be variable, depending on targeted population. In this one, all members of ESGURS are surgeons specialized in US, so our results are not from all European urologists but from those devoted to this challenging pathology. This selection bias should add strength to our results, helping to describe current practice in most of the specialized centres in Europe. In addition, we can learn about use of most recent techniques (i.e., non-transecting) which are not very common among previously surveyed urologists. We used the same questionnaire as previously published to increase comparability, even when some surgical options are not currently of choice, as using skin grafts. Again, we believe genitourinary reconstructive surgeons working in referral centres should be able to offer different techniques for managing complex and difficult cases where standard options are not suitable.

## 5. Conclusions

ESGURS members mainly work in public Teaching-Academic hospitals and consider themselves to have an adequate specific training in US. Referral US units are seen as a need, and both theoretical and hands-on-courses are recommended. RUG is the most common tool for diagnosis and UF for follow-up, with an increasing use of PROM questionnaires. The limit length for DVIU is <2 cm. The so-called “therapeutic ladder” should be avoided and a definitive urethroplasty should be offered if indicated. For bulbar strictures, the use of dorsal skin/oral grafts is the most frequent treatment. New techniques such as “non-transecting” urethroplasties are applied by a significant number of well-trained surgeons.

## Figures and Tables

**Table 1:** Age distribution, hospital type and level.

Question	Options	Nº of Urologists (%)
Age group	30–39	10 (20.4)
	40–49	9 (18.4)
	50–59	19 (38.8)
	≥60	11 (22.5)
Type of practice	Private hospital	7 (14.3)
	Private teaching hospital	5 (10.2)
	Public hospital	4 (8.2)
	Public teaching hospital	33 (67.4)
	Private hospital	7 (14.3)
Hospital level	Rural commune (<5000 inhabitants)	0
	Provincial town (5000–20,000 inhabitants)	1 (2.0)
	Medium-sized city (20,000–100,000) inhabitants	2 (4.1)
	Major city (>100,000 inhabitants)	46 (93.9)

**Table 2:** Country of practice of ESGURS members participants.

Country of Practice	Nº of Urologists
Italy	7
Germany	5
United Kingdom	5
Belgium	4
Russia	4
India	3
Norway	3
Egypt	2
Israel	2
Netherlands	2
Serbia	2
Spain	2
France	1
Turkey	1
Austria	1
Greece	1
Morocco	1
Colombia	1
Sweden	1
Finland	1

**Table 3:** Number of urethral stricture patients and urethroplasties performed last year.

Question	Options	Nº of Urologists (%)
Nº of patients with urethral strictures treated during the last year	None	0 (0)
	1–5	3 (6.1)
	6–10	4 (8.2)
	11–20	6 (12.2)
Nº of urethroplasties performed during the last year	>20	36 (73.5)
	None	3 (6.4)
	1–5	5 (10.6)
	6–10	2 (4.3)
	11–20	11 (23.4)
	>20	26 (55.3)

**Table 4:** Diagnostic and follow-up tests.

Diagnostic Test	Nº of Urologists (%)	
	Diagnostic Work-Out	Follow-Up
Retrograde urethrogram +/− voiding cysto-urethrography	46 (93.9)	27 (57.5)
Uroflowmetry +/− post-void residual	44 (89.8)	45 (95.7)
Urethro-cystoscopy (flexible/rigid)	32 (65.3)	14 (29.8)
Urethral ultrasonography	10 (20.4)	3 (6.4)
Urethral calibration	5 (10.2)	6 (12.8)
IPSS *	16 (32.7)	13 (27.7)
PROM ** urethra	21 (42.9)	20 (42.6)
Other questionnaires (i.e., IIEF ***)	16 (32.7)	13 (27.7)

* IPSS: international prostate symptom score, ** PROM: patient-reported outcomes, *** IIEF: international index on erectile function.

**Table 5:** Therapeutic approaches performed over the last 2 years.

Techniques		Nº of Urologists (%)
Endoscopic procedures	Urethral dilation	31 (63.3)
	Patient intermittent self-dilations/CIC *	28 (57.1)
	Direct vision endoscopic internal urethrotomy (Sachse)	39 (79.6)
	Blind endoscopic internal urethrotomy (Otis)	14 (28.6)
	Laser endoscopic internal urethrotomy	8 (16.3)
	Endo-urethral stent implantation (Memokath, Urolume, Allium)	6 (12.2)
Urethroplasty (open) procedures	External meatotomy	22 (44.9)
	Meatoplasty	42 (85.7)
	End-to-end anastomotic urethroplasty	41 (83.7)
	“Non-transecting” anastomotic urethroplasty	31 (63.4)
	Urethroplasty using skin flaps (preputial, penile, scrotal)	30 (61.2)
	Urethroplasty using grafts (skin, oral mucosa)	45 (91.8)
	Perineal urethrostomy	38 (77.6)

* CIC: Clean intermittent self-catheterization.

**Table 6:** Preferred urethroplasty techniques for bulbar strictures.

Urethroplasty Technique	Nº of Urologists (%)
Urethroplasty using grafts (preputial, oral mucosa) dorsally located	16 (36.4)
End-to-end anastomotic urethroplasty	14 (31.8)
Urethroplasty using grafts (preputial, oral mucosa) ventrally located	13 (29.5)
Urethroplasty using skin flaps (preputial, penile, scrotal)	1 (2.3)

**Table 7:** How would you manage in your clinical practice a 35 year-old male, uncircumcised, with a 3.5 cm idiopathic bulbar urethral stricture, complaining of poor flow and with maximum flow rate of 7 mL/s?

Urethroplasty Technique	Nº of Urologists (%)
Refer the patient to another Urologist from my Hospital	3 (6.4)
Refer the patient to another Hospital	1 (2.1)
Urethral dilation	2 (4.3)
Endoscopic internal urethrotomy (cold knife, laser)	3 (6.4)
Endoscopic internal urethrotomy (cold knife, laser) + patient self-dilations/CIC *	2 (4.3)
End-to-end anastomotic urethroplasty	2 (4.3)
“Non-transecting” anastomotic urethroplasty	1 (2.1)
Urethroplasty using grafts (preputial, oral mucosa) dorsally located	22 (46.8)
Urethroplasty using grafts (preputial, oral mucosa) ventrally located	11 (23.4)

* CIC: clean intermittent self-catheterization.

**Table 8:** How would you manage in your clinical practice a 24 year-old male, with a 1 cm idiopathic proximal bulbar urethral stricture, with 2 previous internal urethrotomies (last one 6 months ago), complaining of poor flow and with maximum flow rate of 6 mL/s?

Urethroplasty Technique	Nº of Urologists (%)
Refer the patient to another Urologist from my Hospital	4 (8.7)
Endoscopic internal urethrotomy	1 (2.2)
Endoscopic internal urethrotomy (cold knife, laser) + patient self-dilations/CIC *	2 (4.4)
End-to-end anastomotic urethroplasty	14 (30.4)
“Non-transecting” anastomotic urethroplasty	11 (23.9)
Urethroplasty using grafts (skin, oral mucosa) dorsally located	5 (10.9)
Urethroplasty using grafts (skin, oral mucosa) ventrally located	9 (19.6)

* CIC: clean intermittent self-catheterization.

## Data Availability

Not applicable.

## Supplementary Materials

The following supporting information can be downloaded at: https://www.mdpi.com/article/10.3390/jcm11092353/s1. Supplementary Material S1: Questionnaire about Practices and Opinions Related with Management of Male Anterior Urethral Strictures.
