# Supplementary material for: Contemporary Management of Male Anterior Urethral Strictures by Reconstructive Urology Experts—Results from an International Survey among ESGURS Members
Source: J Clin Med. 2022 Apr 22;11(9):2353. doi: 10.3390/jcm11092353 (PMC9103897; doi:10.3390/jcm11092353)
Supplement: Supplementary file 1 [file jcm-11-02353-s001.zip › ESGURS survey JCM 1.0 (Supplementary Material).pdf]

Supplementary material S1:

QUESTIONNAIRE ABOUT PRACTICES AND OPINIONS RELATED WITH MANAGEMENT OF MALE ANTERIOR URETHRAL STRICTURES

1) Please state your age group:
   30-39
   40-49
   50-59
   > 60

2) Type of practice:
   Public hospital
   Public teaching/academic hospital
   Private hospital
   Private teaching/academic hospital

3) Level of the hospital where you practice:
   Major city (>100.000 inhabitants)
   Medium-sized city (20,000–100.000 inhabitants)
   Provincial town (5,000–20.000 inhabitants)
   Rural commune (<5.000 inhabitants)

4) Country where you practice (Please select only one option):
   (List of European Association of Urology members´ countries where they practice)

5) Is there a specialized unit/person dedicated to urethral stricture disease in your hospital?:
   Yes
   No

6) Please state the (approximate) number of patients with urethral strictures that you have treated during last year:
   None
   1-5
   6-10
   11-20
   >20

Please, only proceed with the following questions if you routinely manage in your practice patients with urethral strictures and the answer to previous question was different than 1) None

7) During evaluation of anterior urethral strictures, before surgical indication, which diagnostic methods do you usually perform in your routine practice? (Check all that apply)
   Uroflowmetry +/- post-void residual
   Urethral calibration (catheters, sounds, bougies à boule)
   Retrograde urethrogram +/- voiding cysto-uretrography
   Urethral ultrasonography

Urethro-cystoscopy
IPSS (International Prostate Symptom Score)
PROMs-Urethra (Patient Reported Outcome Measure)
Other questionnaires (i.e. IIEF)

8) Which of the following reconstructive procedures have you performed in the last 2 years? (Check all that apply):
Urethral dilation
Patient intermittent self-dilations/self-catheterisations
Direct vision endoscopic internal urethrotomy (Sachse)
Blind endoscopic internal urethrotomy (Otis)
Laser endoscopic internal urethrotomy
Endo-urethral stent implantation (Memokath, Urolume, Allium)
External meatotomy
Meatoplasty
End-to-end anastomotic urethroplasty
“Non-transecting” urethroplasty (end-to-end anastomotic without complete transection)
Urethroplasty using skin flaps (preputial, penile, scrotal)
Urethroplasty using grafts (skin, oral mucosa)
Perineal urethrostomy

9) If you perform internal urethrotomies or urethral dilations, what is the maximal stricture length that you consider suitable for using these techniques?:
< 1 cm
< 1.5 cm
< 2 cm
< 2.5 cm
< 3 cm
More than 3 cm

10) If you perform internal urethrotomies or urethral dilations, do you routinely use a guidewire or ureteral catheter to reference the urethral lumen during the procedure?:
Yes
Only in selected cases
No

11) If you perform internal urethrotomies or urethral dilations, how long do you usually keep the urethral catheter in place after the procedure?:
I do not routinely leave urethral catheter
24 hours
2-3 days
4-6 days
1-3 weeks
More than 3 weeks

12) If you leave a urethral catheter after internal urethrotomies or urethral dilations, which size of catheter do you routinely choose?:
I do not have a preferred size

12 F
14 F
16 F
18 F
20 F
22 F or wider

13) During follow-up of anterior urethral stricture patients, what methods do you use for evaluate the outcomes and detect recurrences? (Check all that apply):
    Uroflowmetry +/- post-void residual
    Urethral calibration (catheters, sounds, bougies à boule)
    Retrograde urethrogram +/- voiding cysto-uretrography
    Urethral ultrasonography
    Urethro-cystoscopy
    IPSS (International Prostate Symptom Score)
    PROMs-Urethra (Patient Reported Outcome Measure)
    Other questionnaires (i.e. IIEF)

14) If you perform retrograde urethrograms during diagnosis and/or follow-up for these patients, who carry out those test?
    Myself (or another Urologist)
    A Radiologist

15) How would you manage the following patient in your clinical practice? 34 year-old male, uncircumcised, with a 3.5 cm idiopathic bulbar urethral stricture, complaining of poor flow and with maximum flow rate of 7 ml/s. (Check only one answer):
    Refer the patient to another Urologist from my Hospital.
    Refer the patient to another Hospital
    Urethral dilation
    Urethral dilation + Patient intermittent self-dilations/self-catheterisations
    Endoscopic internal urethrotomy (cold knife, laser)
    Endoscopic internal urethrotomy (cold knife, laser) + intermittent self-dilations
    Endo-urethral stent implantation (Memokath, Urolume, Allium)
    End-to-end anastomotic urethroplasty
    "Non-transecting" anastomotic urethroplasty
    Urethroplasty using skin flaps (preputial, penile, scrotal)
    Urethroplasty using grafts (skin, oral mucosa, preputial mucosa), dorsally located
    Urethroplasty using grafts (skin, oral mucosa, preputial mucosa), ventrally located

16) How would you manage the following patient in your clinical practice? 24 year-old male, with a 1 cm idiopathic proximal bulbar urethral stricture, with 2 previous DVIU in the last 2 years, complaining of poor flow and with maximum flow rate of 6 ml/s. (Check only one answer):
    Refer the patient to another Urologist from my Hospital.
    Refer the patient to another Hospital
    Urethral dilation
    Urethral dilation + Patient intermittent self-dilations/self-catheterisations

Endoscopic internal urethrotomy (cold knife, laser)
Endoscopic internal urethrotomy (cold knife, laser) + intermittent self-dilations
Urethral stent implantation (Memokath, Urolume, Allium)
End-to-end anastomotic urethroplasty
"Non-transecting" anastomotic urethroplasty
Urethroplasty using skin flaps (preputial, penile, scrotal)
Urethroplasty using grafts (skin, oral mucosa, preputial mucosa), dorsally located
Urethroplasty using grafts (skin, oral mucosa, preputial mucosa), ventrally located

17) Regarding the management of urethral strictures, which policy do you consider as adequate according to current literature?:
    A "therapeutic ladder": starting the treatment using minimally invasive procedures (UD, DVIU) and considering urethroplasty only after repeated failure of these procedures.
    Choose an urethroplasty as primary option, in the cases when indicated

18) Do you consider the creation/maintenance of Referral Units or Centres for the management of male anterior urethral stricture disease necessary?:
    Yes
    No

19) Related with your specific training on management of urethral stricture disease, would you consider it as adequate?
    Yes
    No
20) Do you consider courses and/or workshops on management of urethral stricture disease useful?
    Yes, only theoretical courses
    Yes, only hands-on courses
    Yes, both theoretical and hands-on courses
    No

Please, only proceed with the following 3 questions if you perform urethroplasty surgeries

21) If you perform urethroplasties, how many of them have you performed during last year?
    1-5
    6-10
    11-20
    >20

22) For a bulbar urethroplasty, what is your preferred technique? (Check only one answer):
    End-to-end anastomotic urethroplasty.
    Urethroplasty using grafts (skin, oral mucosa) ventrally located
    Urethroplasty using grafts (skin, oral mucosa) dorsally located
    Urethroplasty using skin flaps (preputial, penile, scrotal)

23) If you perform urethroplasties, do you routinely perform radiographic test before removal of urethral catheter?:

No, I remove the catheter without imaging testing.
I do not use these tests routinely, only depending on each case.
Yes, I routinely perform imaging checks before of immediately after catheter removal.
